# Insanitary Pavements

**Published:** 1899-10-14

**Authors:** 


### INSANITARY PAVEMENTS.

The Marylebone Vestry, a body which under the influence of recent legislation will before long be superseded by some form of municipal government differing at least in name from its predecessor, appears to have determined upon concluding its career by such an evidence of unfitness for its duties as may be furnished by one crowning act of incapacity. It has commenced paving the long-neglected surface of Harley Street with wood, very much in the manner which Sydney Smith once said would be almost universally practicable, if the local authorities would only "lay their heads together." The wood in question is now piled on the sides of the roadway, where it may be seen to consist of roughly-sawn blocks of soft deal, wholly unprepared to resist the corroding influences of moisture and of organic matter, with both of which, as soon as it is placed *in situ*, it must speedily become saturated. Harley Street, we need scarcely say, is largely tenanted by members of the medical profession; but the preparations for the outrage we are describing had made considerable progress while many of them were still absent on their autumnal holidays. Those who remained, or who had already returned, lost no time in the preparation of a memorial to the vestry, in which it was pointed out that the soft and unprepared wood would speedily be worn into holes, the constantly-recurring cost of repairing which would more than compensate for any original cheapness; and that the abundant dust which would arise in dry weather would be of the most noxious description, and certain to produce ophthalmia, sore throats, and other affections of the mucous membranes, alike in the persons of residents and of passers-by. In the course of a single day, this memorial was not only numerously signed, but some of the residents, dissatisfied with the mild character of the expostulation which it conveyed, appended remarks more or less pungent to the document originally submitted to them. If a consensus of medical opinion could have any influence with the "porochial" authorities, there can be no doubt that the threatened plague would be stayed; but it is much to be feared that "porochialism" will be too strong alike for science and for common sense. The folly capable of entertaining the scheme which is protested against is of that precise character which may be expected to be also capable of persevering in it.

The moral of the whole transaction is not far to seek. It is that the powers of local authorities to inflict permanent mischief should be placed under some control from which they are at present free. It is all very well to say that the authorities in question are elected by the ratepayers, and that these must bear the consequences of an unwise choice of their representatives. Even if the delinquents were turned out at the next election, the pavement would remain; and the pavement of Harley Street affects numbers of people who are neither residents nor ratepayers in the parish. What is manifestly wanted is that there should exist some permanent committee of reference in regard to the various matters of a highly technical nature which are constantly coming before the several vestries and other boards, so that on the one hand these bodies should not be constantly trying experiments in paving and lighting and other matters on the unfortunate residents and ratepayers; and on the other, that something like uniformity should be maintained in the treatment of the streets in contiguous parishes.

